# Unique Opportunities and Challenges From Two Online Psychosocial Randomized Clinical Trials

**DOI:** 10.1093/geroni/igab046.313

**Published:** 2021-12-17

**Authors:** Carol Musil, Britney Webster, Rachel Pruchno

## Abstract

Research aimed at testing readily delivered online psychosocial interventions for addressing the needs of custodial grandfamilies (CGF) has been scarce. This symposium reports on two NIH-funded randomized clinical trials (RCT) involving fully online interventions: Study 1 (S1)-dyadic social Intelligence training for custodial grandmothers and their adolescent grandchildren, and Study 2 (S2)-4 week resourcefulness training with daily journaling intervention for grandmothers only. We presented here on the unique advantages and challenges of online RCTs as they apply to CGFs and similar hard-to-reach populations. First, in a cross-study collaboration, Jeanblanc et al. report data from both studies on how COVID-19 influenced coping habits, grandchild’s remote learning, household conflict, uncertainty, and finances. Second, Castro et al. investigate how baseline positive and negative affect were reported in daily diaries completed by both grandmothers and grandchildren across 14 days at pretest in S1. Third, Musil et al. describe the challenges and benefits of using an entirely online design for the distribution and collection of longitudinal data, as exemplified by 4 weeks of qualitative daily journals from S2. Lastly, Webster et al. report on the benefits and challenges of recruitment and retention strategies encountered across S1 and S2. As discussant, Rachel Pruchno considers how the specific methodological advantages and disadvantages of online RCTs covered in the above papers apply to family caregiving research in general.

